# The Diagnosis between Pyloric Colic and Biliary Colic

**Published:** 1909-03-06

**Authors:** 


### THE DIAGNOSIS BETWEEN PYLORIC COLIC AND BILIARY COLIC.

The possibility of mistaking pyloric colic for biliary colic is particularly great in those cases in which the characteristic evidences of pyloric stenosis, such as gastric rigidity, very copious vomiting, and so forth are absent, or in which jaundice appears as a result of secondary duodenal catarrh (Schmidt*). Sometimes, though fortunately rarely, the two conditions occur in combination. Some of the more important differential signs may be summarised in the following table:

| Pyloric Colic. | Biliary Colic. |
|---|---|
| Active borborygmi in the epigastrium. | |
| Distension most marked below the left costal border. | Swelling in the gall-bladder region. |
| Acid eructations with heartburn; copious vomiting of strongly acid material that is not bile-stained but contains sarcinæ and possibly particles of old food. | Vomiting of bitter material that is bile-stained and is not very great in amount. |
| Eructations smelling of $H_2S$. | |
| Copious vomiting or eructations of gas are followed by a marked diminution in the pain. | Vomiting has no noteworthy effect on the pain, or it may even increase it. |
| Usually no chill. | Often a chill followed by elevation of temperature. |
| The fasting stomach contains old food. | |
| Attacks are very numerous, often occurring daily for weeks or months. | Attacks are sporadic, frequently with intervals of several months. |
| The pain tends to radiate to the left. | The pain tends to radiate to the right. |
| The attacks regularly begin two to three hours after the midday or largest meal. | Irregularity in time of onset or a longer interval after eating—about five hours. |
| Foods causing gas-formation tend to increase the pain. | The nature of the food is of comparatively slight effect. |
| Attacks of colic are sometimes brought on by lying on the right side. | The left lateral position is often badly borne, and is accompanied by a feeling of painful traction on the right. |
| Local anæsthetics relieve the pain. | |
| | The urine contains bilirubin or urobilinogen. |

Numerous as the differential signs are, it may in some cases be exceedingly difficult to distinguish between these widely separated pathological conditions. On the one hand there are cases of very slight pyloric stenosis, in which there is good compensation and the objective cardinal symptoms are absent or few; but in which, possibly in consequence of general irritability of the nervous system, the attacks of pain may be extremely severe. While, on the other hand, cholelithiasis may be accompanied by symptoms such as gastralgia or pain due to adhesions between gall-bladder and duodenum, which arouse the suspicion of a pyloric stenosis due to ulceration. Finally, of course, the two conditions may co-exist.

* Schmidt, Rudolph: "Pain." See review in The Hospital of Nov. 21, 1908, p. 201.

